# How improvisation drives lean search: The moderating role of entrepreneurial team heterogeneity and environmental uncertainty

**DOI:** 10.3389/fpsyg.2022.940273

**Published:** 2022-09-29

**Authors:** Bo Huang, Jianmin Song, Yanguo Jing, Yi Xie, Yuyu Li

**Affiliations:** ^1^School of Economics and Business Administration, Chongqing University, Chongqing, China; ^2^Faculty of Business, Computing and Digital Industries, Leeds Trinity University, Coventry, United Kingdom; ^3^School of Economics and Management, Wuhan University, Wuhan, China; ^4^School of Economics and Management, Chongqing Normal University, Chongqing, China

**Keywords:** exploitation, exploration, improvisation, team heterogeneity, lean startup

## Abstract

Although lean search is seen as an important action in lean startup, previous studies have less knowledge on how to realize it, especially in the face of traditional plans that cannot cope with sudden changes in the environment. To fill the research gap, this study investigates the effects of improvisation (exploitative, explorative, and ambidextrous improvisation) on lean search. Meanwhile, this research also discusses the moderating effects of entrepreneurial team heterogeneity and the environmental uncertainty to identify the boundary conditions of this relationship. Supported by the cross-sectional data from 203 Chinese startups, the results show that explorative and ambidextrous improvisation are positively associated with lean search. However, the effect of exploitative improvisation on lean search is unsupported. Additionally, technology uncertainty positively moderates the relationship between exploitative improvisation and lean search. Market uncertainty positively moderates the relationship between explorative improvisation and lean search. However, the entrepreneurial team heterogeneity negatively moderates the relationship between ambidextrous improvisation and lean search. These findings contribute to understanding how startups could conduct lean search in a rapidly changing environment, which provides theoretical guidance for improving the success rate of startups.

## Introduction

Recently, a business report published by the *Wall Street Journal* reveals that over 70% of startups fail to survive (Gage, 2012; Yu and Wang, 2021). This occurs because startups do not test their business models and products in the market before launching, unlike mature enterprises (Blank, 2013; Ghezzi and Cavallo, 2020). To solve this problem, many startups have adopted the *lean startup* method to improve entrepreneurial success (Blank, 2013; Shepherd and Gruber, 2021). The lean startup method is an entrepreneurial approach that promotes new product development and business model validation by linking customer feedback with minimum viable products (MVP; Ries, 2011; Felin et al., 2020). In this process, startups need to first search for a reasonable and profitable product and business model. The marketing activities of the firm and scaling up should only be executed when the customer demands are verified and satisfied in the research stage (Yang et al., 2019). Thus, the lean search stage usually reflects the core ideas of the lean startup better than the lean execution.

Although the lean search stage is an important part of the lean startup, previous studies did not know how to perform it (Blank, 2013; Silva et al., 2020; Shepherd and Gruber, 2021; Zhu and Dong, 2021). More importantly, they neglect the fact that there is a paradox in lean search, i.e., product iteration and customer validation are time-consuming, while the rapid change in the environment requires them to perform these activities in a relatively short time. Therefore, the problem of realizing lean search in a rapidly changing environment needs to be solved urgently. Second, earlier studies pay less attention to the boundary conditions under which a lean search is conducted (Yang et al., 2019; Zhu and Dong, 2021). Therefore, they fail to understand when the strength of the lean search increases or decreases. Zhu and Dong (2021) particularly suggest in their study that a promising future direction for lean startup research is the determination of the moderating effects of the external environment and the internal context.

To fill these two research gaps, the study identifies the important antecedent factors and moderator variables of lean search in a rapidly changing context. Essentially, unlike mature firms with larger resource bases, startups prefer to take advantage of improvisation to deal with the time paradox of implementing lean search. This is because improvisation emphasizes the spontaneous integration and utilization of existing resources in a short span of time (Hmieleski and Corbett, 2006; Ye et al., 2018; Prashantham and Floyd, 2019). Some studies also show that startups which embrace improvisation are more likely to utilize instantaneous opportunities and fast learning to cross the threshold of survival (Hu et al., 2018; Liu et al., 2018; Prashantham and Floyd, 2019; Xiong et al., 2019). Thus, improvisation might motivate startups to conduct lean search activities. However, few studies have investigated this relationship by linking improvisation with lean search.

Additionally, both improvisation and lean search are influenced by the internal and external environment (Ye et al., 2018; Mansoori et al., 2019). On the one side, environmental uncertainty is an exogenous factor that influences entrepreneurial activities (Hadida and Tarvainen, 2015; Li et al., 2020). On the other side, the characteristics of team members (e.g., heterogeneity) also play an important role in the organization. This is because most entrepreneurial activities are conducted by teams with complementary strengths (Dufays and Huybrechts, 2016; Kier and McMullen, 2018). Thus, environmental uncertainty and entrepreneurial team heterogeneity are essential conditions that influence the relationship between improvisation and lean search. Unfortunately, previous studies did not integrate these conditions into the analytical framework of lean search.

Based on these arguments, this study aims to answer two related questions: RQ1: Does improvisation foster lean search? RQ2: To what extent do entrepreneurial team heterogeneity and environmental uncertainty influence the relationship between improvisation and lean search? In addressing these two questions, a theoretical model is established in the study. First, improvisation is taken as the antecedent variable and could be of two types, i.e., exploitative and explorative improvisation (Ye et al., 2018; Xiong et al., 2019). Second, the dependent variable in this theoretical model is the lean search, which focuses on customer exploration and validation (Yang et al., 2019; Ghezzi and Cavallo, 2020). Third, entrepreneurial team heterogeneity and environmental uncertainty are the two moderators involved in this theoretical framework. Fourth, the study draws on the cross-sectional survey data from 203 Chinese startups and empirically adopts structural equation modeling in order to support our hypotheses.

Twofold theoretical contributions generate in this study. Primarily, the study fills the gap that was neglected by previous studies and the discussion of the antecedent factors of lean search. As mentioned in the study by Silva et al. (2020), mainstream studies know so much about the benefits of the lean startup in detail. This includes improving entrepreneurial performance (Harms, 2015; Yang et al., 2019). However, information on ways to perform a lean search is limited. By testing the causal relationship between improvisation and lean search, the study provides theoretical evidence for determining how startups conduct a lean search in a short time. Secondly, the study also narrows the gap that the previous studies ignored, i.e., ignorance of the boundary conditions for conducting a lean search. By empirically testing the moderating results of entrepreneurial team heterogeneity and environmental uncertainty, the research contributes to answering the question “when the lean search gets stronger or weaker.” Thus, the study provides a comprehensive understanding on matching the different types of improvisation and different internal and external conditions in order to foster lean search.

The rest of the study is presented in five parts. The conceptions of core constructs and theoretical framework are presented in section Theoretical background. We develop the research hypotheses in section Hypotheses development. Section Research methods describes the research methods, including the sample and measurement. The results are reported in section Results. Section Discussion and conclusion illustrates the findings, the theoretical and managerial implications, the limitations, and future directions.

## Theoretical background

### Improvisation and its ambidexterity

Improvisation mainly originates from jazz and theater. The actors had to randomly adjust and improvise their performances according to the atmosphere of the scene (Moorman and Miner, 1998). Later, improvisation was introduced into the field of organization which mainly reflected the response of enterprises to emergencies (Vera and Crossan, 2005; Ye and Mai, 2018). Mainstream studies have extensively discussed related issues from the point of improvisation. For example, a high level of improvisation leads to high performance (Hmieleski and Corbett, 2008; Hadida and Tarvainen, 2015). However, the studies overlooked the different forms of improvisation (Cunha et al., 2003; Ye et al., 2018).

In fact, startups not only pay attention to efficient allocation and optimization of existing resources to make quick decisions (Cunha et al., 2003; Ye and Mai, 2018) but also emphasize the acquisition and innovation of new knowledge and new resources when facing environmental shocks (Vera and Crossan, 2005). Thus, improvisation in an entrepreneurial context shows the unity of opposites between routine and innovation, and between a structured program and a random combination, which is full of ambidexterity (Cunha et al., 2003; Best and Gooderham, 2015; Hadida and Tarvainen, 2015; Ye and Mai, 2018; Xiong et al., 2019). Our study is based on the findings of Ye and Mai (2018) and focuses on two forms of improvisation from an ambidextrous perspective, including exploitive and the explorative improvisation. Exploitive improvisation is the operational ability to utilize, develop, and optimize existing resources efficiently, rapidly, and spontaneously. Explorative improvisation reflects the creative potential to expand, reorganize, and innovate with new knowledge, resources, and readily available solutions.

### Lean search

Lean search is an important action in lean startup (Ries, 2011; Blank, 2013; Wang et al., 2017; Shepherd and Gruber, 2021). Ries (2011) suggested that startups should not invest a lot of resources in driving customer growth when the value proposition of the new products and business model has not been proven in the market. Alternatively, startups should introduce a minimum viable product (MVP) into the market and adopt a “develop-test-learning” feedback loop which can help in identifying the demands of angel customers, and thus, optimize the MVPs (Harms, 2015; Yang et al., 2019; Silva et al., 2020). Therefore, the lean search is conducted to develop and interact with customers and determine the possible pain points of angel customers (Blank, 2013). By conducting a lean search, startups can improve at listening, observing, and asking potential users for their opinions on the features of the product, pricing, and the available distribution channels to further clarify the direction of the development of the product (Shepherd and Gruber, 2021). Thus, startups can quickly assemble minimized products and iterate and optimize them based on the user pain points to achieve a repeatable, sustainable, and profitable business model (Blank and Dorf, 2012; Mansoori et al., 2019).

### Theoretical framework

The study is to answer the question of “How to realize lean search in a rapid time.” For this, we develop a theoretical model about the effect of improvisation on lean search. First, although the lean search is one of the most important entrepreneurial activities in lean startup, the feedback loop of “product development-customer demand” involved in it is generally long-lasting (Ries, 2011; Blank, 2013). A continuously volatile environment requires startups to take business actions rapidly, which might create a paradox (Ye and Mai, 2018; Yu et al., 2020). Therefore, improvisation is the preferred strategy during environmental changes (Hadida and Tarvainen, 2015). Second, improvisation emphasizes the acquisition of new resources along with the efficient use of current resources while using available resources in response to environmental changes (Xiong et al., 2019). This suggests that improvisation is full of ambidexterity, which can be grouped into exploitative and explorative improvisation (Cunha et al., 2003; Ye and Mai, 2018).

**Figure 1:**
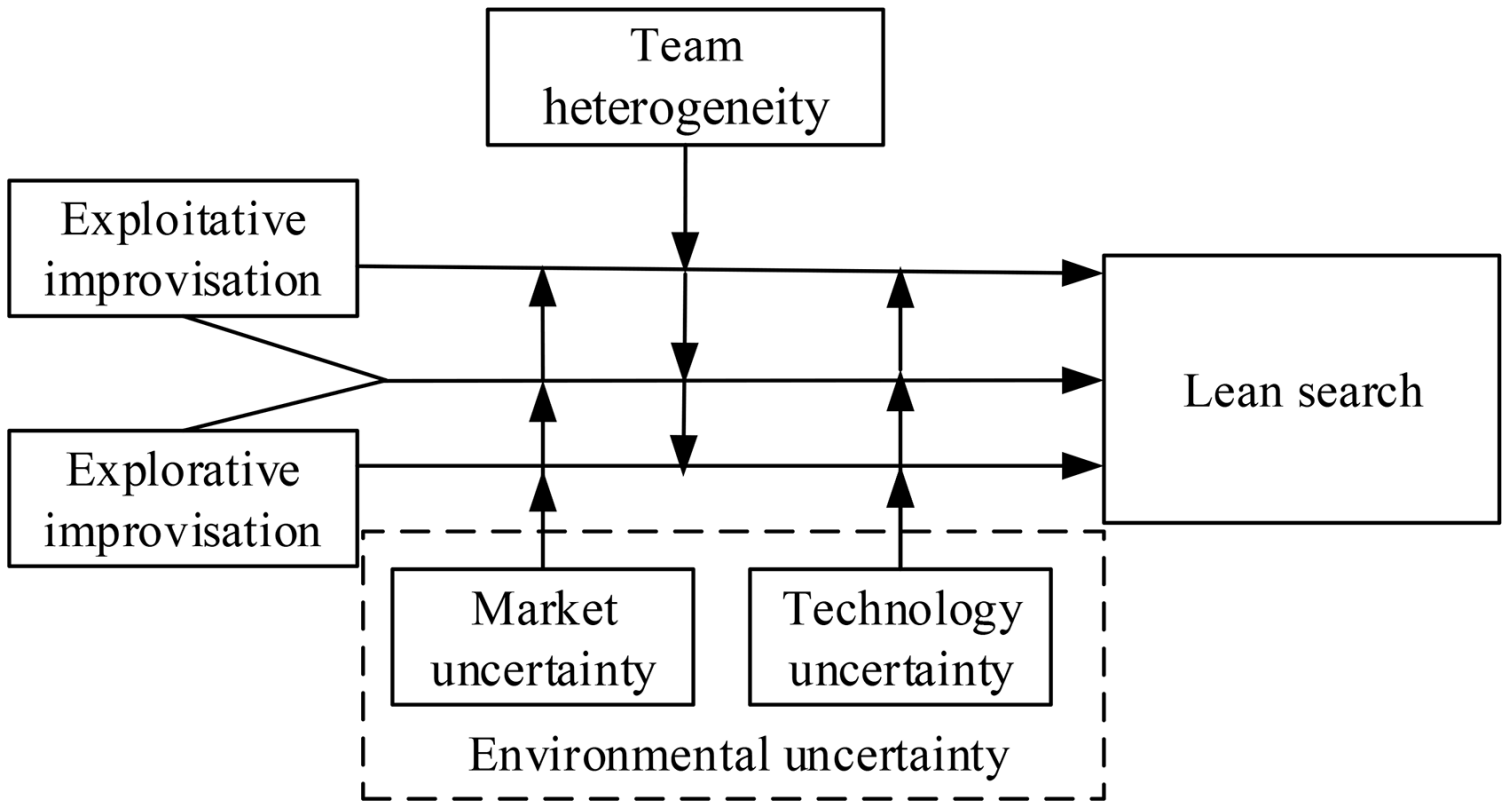
Theoretical framework.

Third, the theory of environment alignment suggests that the entrepreneurial team heterogeneity and the environmental uncertainty are deemed important internal and external conditions (Kier and McMullen, 2018; Li et al., 2020). The entrepreneurial team heterogeneity, as the level of differentiation among team members regarding cognitive concepts, experiences, attitudes, and demographic characteristics, contributes to the reduction in the asymmetry of information and the development of more innovative ideas (Hu et al., 2014; Dufays and Huybrechts, 2016; Leitch and Volery, 2017). Thus, it can influence the relationship between improvisation and lean search (Heyden et al., 2013; Ma et al., 2021). In addition, the threefold marketization, globalization, and decentralization characterize the entrepreneurial environment in China (He et al., 2019). Compared to the developed economies, entrepreneurial activities in China have more market opportunities. However, their potential threats are from customer demands, competitors, and the technological revolution (Li et al., 2020). Thus, the external environment is uncertain regarding markets and technology (Zhang et al., 2020). Based on the above arguments, the study develops the following research framework in Figure 1.

## Hypotheses development

### Improvisation and lean search

Since startups generally have a high degree of the “liability of newness,” they focus more on the spontaneous response to environmental changes than mature enterprises (Yu et al., 2020). Thus, startups are more likely to improvise strategies for conducting entrepreneurial activities (Hmieleski and Corbett, 2006; Valaei et al., 2017; Hu et al., 2018; Prashantham and Floyd, 2019). Improvisation can accelerate the reallocation of resources by enabling startups to seize instant opportunities and reduce the learning time (Liu et al., 2018). In this process, exploitative improvisation can facilitate the efficient use of available resources to accelerate the development of MVPs. This can help startups to discover and respond to the demands of their angel customers (Mihalache et al., 2014; Ye and Mai, 2018). Exploitative improvisation accelerates the effectiveness and clarifies the direction of product development. Thus, a new business model can be validated quickly (Lyles et al., 2014; Yang et al., 2019). Additionally, exploitative improvisation also greatly reduces the cycle time significantly to allow startups to exploit the market opportunities (Ye and Mai, 2018; Xiong et al., 2019). Through this process, startups can better understand their objectives and can accurately implement activities, such as “customer lock-in” and “customer expansion” (Tuan, 2016; Ye and Mai, 2018). Based on these arguments, we propose a hypothesis as follows:

*H1*: Exploitative improvisation is positively associated with lean search.

Additionally, the verification of early customers by alleviating resource constraints and promoting organizational diversity can be facilitated by explorative improvisation. This is because explorative improvisation emphasizes experimentation, innovation, and risk-taking of new resources that are readily available (Xiong et al., 2019; Felin et al., 2020). Explorative improvisation increases the attempts to combine various new resources, which helps to expand the customer search (Ries, 2011). More importantly, it helps startups to develop and validate sustainable business models and new products to diversify their demands (Yang et al., 2019). For startups, meeting customer demands with MVPs is challenging, several iterations of products and demands are required (Shepherd and Gruber, 2021). Thus, explorative improvisation not only effectively shortens the information gap between products and customers and improves customer stickiness from a customer-oriented perspective but also encourages startups to pay more attention to these activities, such as “customer search” and “business model verification” (Xiong et al., 2019; Shepherd and Gruber, 2021). Based on these arguments, we propose a hypothesis as follows:

*H2*: Explorative improvisation is positively associated with lean search.

Although both exploitative and explorative improvisation can promote lean search, startups might still fall into speculative traps if they overemphasize one single type of improvisation (Valaei et al., 2017; Prashantham and Floyd, 2019). Xiong et al. (2019) proposed that the negative effects of improvisation were not limited to contexts. Instead, the type of dislocation caused by the ambidextrous imbalance is a key reason for low performance. Maintaining the ambidextrous balance of improvisation is the key to stimulating the positive effects of exploitative and explorative improvisation (Valaei et al., 2017; Ye and Mai, 2018). Based on these arguments, the study hypothesizes that ambidextrous improvisation is positively related to lean search.

On the one hand, explorative improvisation fills the resource shortsightedness brought by exploitative improvisation (Xiong et al., 2019). On the other hand, the effectiveness emphasized by exploitative improvisation can also help in avoiding the vicious cycle of innovation, which is a result of explorative improvisation. Under the synergistic effect of explorative and exploitative improvisation, startups can plan future directions and resource allocation methods from a holistic perspective and orient them toward specific development paths, which can increase the success rate of “customer identification” and “customer verification” (Blank, 2013; Ye and Mai, 2018). Additionally, the flow, integration, and innovation of internal and external knowledge are accelerated by ambidextrous improvisation. These drive startups to constantly choose and optimize knowledge combination methods (Ye et al., 2018). This process greatly promotes the activities associated with “product lock-in,” “customer demands lock-in,” and “business model validation.” Therefore, we propose a hypothesis as follows:

*H3*: Ambidextrous improvisation is positively associated with lean search.

### The moderating effect of entrepreneurial team heterogeneity

Due to the absence of formal institutional support, startups rely on entrepreneurial teams to conduct business activities (Kier and McMullen, 2018; Li et al., 2020). Entrepreneurial team heterogeneity refers to the differences in the gender, age, educational background, cognition, and experience of the team members. It is one of the most important organizational contexts in startups (Hu et al., 2014; Leitch and Volery, 2017). Li et al. (2020) showed that entrepreneurial team heterogeneity allows startups to grow by providing various solutions and improving the sharing of knowledge.

While conducting a lean search, high levels of entrepreneurial team heterogeneity can help in minimizing the decision risk in the transient decision process because more comprehensive information can be obtained from diverse industry experience, intellectual assets, and analytical perspectives (Li et al., 2020). It can make the utilization of existing resources more efficient and facilitate the acquisition and combination of new resources. More importantly, a high level of cognitive conflict is brought about by heterogeneous entrepreneurial teams. This brings various innovative ideas and schemes to startups in resource utilization (Hu et al., 2014). Thus, high levels of entrepreneurial team heterogeneity help in stimulating the breadth of resource patching, optimization, development and knowledge reorganization, and the degree of program innovation. This plays an important role in encouraging startups to develop products based on customer demands (Williams, 2016; Li et al., 2020). Moreover, the contradiction between explorative and exploitative improvisation can be effectively alleviated by entrepreneurial team heterogeneity, which can promote the coordination and complementarity of the two. For example, the older team members are more inclined toward using their social networks to expand the market. On the other hand, the younger team members prefer an aggressive risk-taking approach (Hu et al., 2014; Shepherd and Gruber, 2021). Thus, it is proposed that:

*H4a*: Entrepreneurial team heterogeneity positively moderates the relationship between exploitative improvisation and lean search;

*H4b*: Entrepreneurial team heterogeneity positively moderates the relationship between explorative improvisation and lean search;

*H4c*: Entrepreneurial team heterogeneity positively moderates the relationship between ambidextrous improvisation and lean search.

### The moderating effect of environmental uncertainty

Previous studies have stated that improvisation and a high dynamic environment are closely associated (Hmieleski and Corbett, 2006; Ghezzi and Cavallo, 2020). Chandler et al. (2011) pointed out in the research that new ventures under high uncertainty pay greater attention to resource allocation in entrepreneurial actions. Regarding this, the study hypothesizes that the relationship between improvisation and lean search might be strengthened by environmental uncertainty. Also, we focus on the two types of environmental uncertainty: market uncertainty and technology uncertainty (Jiang and Ma, 2018).

Market uncertainty is the unstable state of customer demand preferences, which makes it more challenging for startups to grasp the changing rules of customer demands (Jaworski and Kohli, 1993). Under these circumstances, startups have to focus more on finding ways to better cope with environmental shocks to overcome the survival trap by spontaneously and rapidly exploring potential market opportunities (Foss et al., 2019; He et al., 2019; Li et al., 2020). Thus, startups increase improvisation to capture angel customers more quickly. For example, they speed up the customer exploration and validation process (Hmieleski and Corbett, 2006; Felin et al., 2020). Based on these arguments, it is proposed that:

*H5a*: Market uncertainty positively moderates the relationship between exploitative improvisation and lean search;

*H5b*: Market uncertainty positively moderates the relationship between explorative improvisation and lean search;

*H5c*: Market uncertainty positively moderates the relationship between ambidextrous improvisation and lean search.

Technology uncertainty reflects the degree of technological changes. High levels of technology uncertainty require startups to pay more attention to situational innovations, which are triggered by improvisation (Koka et al., 2006; Ghezzi and Cavallo, 2020). This means that startups can improve the lock-in of angel customers to accelerate the iteration of products and business models. This can be done by shaping new product value propositions. Furthermore, technological turmoil has also created blue ocean markets. These are more conducive to startups in the process of rapid response to the increase in mining customer needs (He et al., 2019). For example, customers generally expand their search for information on product features, pricing, and distribution channels by listening, observing, and asking. Based on these arguments, it is proposed that:

*H6a*: Technology uncertainty positively moderates the relationship between exploitative improvisation and lean search;

*H6b*: Technology uncertainty positively moderates the relationship between explorative improvisation and lean search;

*H6c*: Technology uncertainty positively moderates the relationship between ambidextrous improvisation and lean search.

## Research methods

### Data and sample

To test the hypotheses, the research employed the self-administered survey method to collect cross-sectional data in China. There were a few reasons to use this method. First, this method had some advantages in exploratory research and prediction theory testing and was well-suited to the research status of lean search and improvisation (Straub et al., 2004). Second, all core constructs in the study were developed from previous studies. However, they could not be measured accurately by secondary objective indicators. However, the survey method could make up for the defects in the measurements. Third, the limited public disclosures of startups restricted the avenues for academics to discuss issues related to organizational strategies. Additionally, a survey method was a suitable method of acquiring first-hand information.

Before distributing questionnaires, startups were defined as enterprises established within 8 years, based on the criteria proposed in previous studies (Gartner and B., 1989; Parida et al., 2016). Additionally, to ensure the consistency of the research object with the research topic, we determined whether the startups performed a lean search by asking the question whether they regularly visited customers to optimize the products. Moreover, instead of selecting MBA or EMBA students, the data were collected by a professional market research company. This method was preferred to obtain valid data in emerging economies (Yang et al., 2012; Wang et al., 2020). The data collection process was described as follows:

First, the sample startups that met the standards from the database of the market company were randomly selected through telephone calls and email exchanges. The purpose of the study was thoroughly explained to reduce the social desirability bias. Using this method, 814 startups were short-listed to participate in the survey. Second, the questionnaire to be filled out was given to the CEO, the senior manager, or the department manager of the startup because they had a good understanding of business models, products, and daily activities (Yuan et al., 2021). Third, the data were collected through telephone interviews and electronic questionnaires. All participants were anonymous. Fourth, the quality of data in the study was ensured by deleting invalid questionnaires with ambiguous and incomplete answers. The questionnaires with short answering times were excluded.

After applying these criteria, 203 valid responses (the valid response rate was 24.94%) were collected. The details of the samples are provided in Table 1. Additionally, we conducted an ANOVA and found no significant differences. Furthermore, we adopted Harman’s single-factor procedure to test the common method variance (CMV) in the study (Podsakoff et al., 2012), and found that the cumulative variance contribution rate of the first factor without the rotation factor was 37%, indicating that there was no substantial CMV. Additionally, we employed another procedure to test CMV proposed by Lisboa et al. (2011). The results showed that *χ^2^* = 1508.887, *df* = 209, *χ^2^/df* = 7.220, *CFI* = 0.526, *TLI* = 0.477, *RMSEA* = 0.175, and *SRMR* = 0.144 were higher than the threshold, indicating that the single-factor model fit was poor, thus CMV was in the acceptable range.

**Table 1:** Descriptive statistics for samples (*N* = 203).

Indexes	Category	Frequency	Per (%)	Indexes	Category	Frequency	Per (%)
Firm size (number of employees)	<10	66	32.51%	Industry	Biotechnology	10	4.93%
	10–50	86	42.36%		Manufacturing	80	39.41%
	51–100	26	12.81%		IT	109	53.69%	
	>100	25	12.32%			Others	4	1.97%

### Measurements

The measurements obtained were operationalized by following previously validated measurement tools and following the translation and back translation procedure proposed by Brislin (1980) to translate them into Chinese versions. Unless the context stated otherwise, all the items were scored on a seven-point Likert-type scale (1 = “*strongly disagree*, 7 = “*strongly agree*”). The measurement scales were presented in Supplementary material.

#### Improvisation

Based on the pioneering study by Ye and Mai (2018), both five-item scales were used to measure exploitative and explorative improvisation. A sample item was “Our company rapidly integrates the past experience to response action.” The reliability and validity were ensured by first conducting CFA (Confirmatory Factor Analysis) on key latent variables to delete the low factor loading (Table 2).

**Table 2:** Reliability and convergent validity test results (*N* = 203).

	Items	CITC	SFL	SMC	α	CR	AVE		Items	CITC	SFL	SMC	α	CR	AVE
EPI	EPI01	0.818	0.861	0.741	0.919	0.920	0.697	EPR	EPR02	0.696	0.782	0.612	0.840	0.840	0.568
	EPI02	0.826	0.871	0.759					EPR03	0.659	0.741	0.549			
	EPI03	0.718	0.752	0.566					EPR04	0.683	0.762	0.581			
	EPI04	0.795	0.845	0.714					EPR05	0.653	0.729	0.531			
	EPI05	0.807	0.841	0.707				*χ^2^/df = 2.447, CFI = 0.991, TLI = 0.972, RMSEA = 0.084, SRMR = 0.018*							
*χ^2^/df = 2.900, CFI = 0.987, TLI = 0.973, RMSEA = 0.097, SRMR = 0.019*								TU	TU01	0.550	0.627	0.393	0.777	0.782	0.547
TH	TH01	0.763	0.794	0.630	0.920	0.922	0.747		TU02	0.659	0.816	0.666			
	TH03	0.862	0.916	0.839					TU03	0.635	0.763	0.582			
	TH04	0.875	0.933	0.870				*χ^2^/df = 1.000, CFI = 1.000, TLI = 1.000, RMSEA = 0.000, SRMR = 0.000*							
	TH05	0.769	0.806	0.650				LS	LS02	0.578	0.728	0.530	0.736	0.737	0.484
*χ^2^/df = 2.621, CFI = 0.995, TLI = 0.985, RMSEA = 0.089, SRMR = 0.010*															
MU	MU01	0.582	0.652	0.425	0.803	0.807	0.585		LS03	0.578	0.722	0.521			
	MU02	0.685	0.815	0.664											
	MU03	0.684	0.815	0.664					LS05	0.525	0.633	0.401			
*χ^2^/df = 1.000, CFI = 1.000, TLI = 1.000, RMSEA = 0.000, SRMR = 0.000*								*χ^2^/df = 1.000, CFI = 1.000, TLI = 1.000, RMSEA = 0.000, SRMR = 0.000*							

EPI, exploitative improvisation; EPR, explorative improvisation; TU, technology uncertainty; MU, market uncertainty; TH, entrepreneurial team heterogeneity; LS, lean search; SFL, Standardized factor loading; α, Cronbach’s Alpha; C.R, Composite reliability; CITC, Corrected item-total correlation; SMC, Square multiple correlations; AVE, Average variance extracted; we excluded EPR01, TH02, LS01, LS04, LS06.

#### Lean search

We used a six-item scale to measure lean search developed by Yang et al. (2019). One of the sample items was “The marketing strategy of our firm is not immutable. On the contrary, it is very flexible.” We conducted CFA and excluded low factor loadings (Table 2).

#### Entrepreneurial team heterogeneity

Based on Naranjo-Gil (2009) and Talke et al. (2010), we used a five-item scale to measure this construct. A sample item was “There is a wide age gap among members of management teams.” CFA was performed to analyze the data (Table 2).

#### Environment uncertainty

We used both three-item scales to measure market uncertainty and technology uncertainty developed by Jaworski and Kohli (1993) and Jiang and Ma (2018). A sample item was “Customer demands change rapidly.” The results of the CFA are shown in Table 2.

#### Control variables

We selected firm type, industry, firm age, and firm size as control variables, following previous studies (Vorhies and Harker, 2000; Smolka et al., 2018). Specifically, we introduced a categorical variable to represent the age of the firm (1 = “within 1 year”; 2 = “1 to 4 years”; 3 = “4 to 8 years”). We also used the number of employees to measure the firm size, which was considered to be a continuous variable (1 = “less than 10 people; 2 = “10 to 50 people”; 3 = “51 to 100 people”; 4 = “more than 100 people”).

## Results

### Validity and reliability

The study used four indices to comprehensively assess the reliability. These indices were Cronbach’s alpha, composite reliability (CR), square multiple correlations (SMC), and corrected item-total correlation (CITC). The results revealed that all Cronbach’s alpha values and the CR of key constructs were higher than the threshold value of 0.7 (Table 2; Fornell and Larcker, 1981). Additionally, the rule of thumb for CITC is recommended to be at least 0.4. Here, all the values of CITC satisfied the principle for all the items. Finally, the SMC of all items was greater than the recommended value of 0.36. These results indicated that all the constructs and each item had good reliability.

To assess the convergent validity, standard factor loading (SFL) was introduced. The results showed that the SFL of all the items was greater than the recommended value of 0.6 (Hair et al., 2011). Also, a rule of thumb for AVE is recommended to be 0.5, and the results demonstrated that exploitative improvisation (AVE = 0.697), explorative improvisation (AVE = 0.568), entrepreneurial team heterogeneity (AVE = 0.747), market uncertainty (AVE = 0.585), and technology uncertainty (AVE = 0.547) exceeded the standard AVE value, but lean search (AVE = 0.484) did not. However, the AVE value of lean search was greater than 0.36, which satisfied the lower bound criterion. These results suggested that the convergence validity of the core blocks presented was acceptable.

In addition, the Fornell–Larcker criterion was adopted to assess the discriminate validity. According to this criterion, the square root of the AVE of each variable should be higher than the correlation coefficient of the row and column in which the variables were located (Fornell and Larcker, 1981). The results showed that (Table 3) the square root of the AVE value of other constructs was greater than the correlation coefficient of the row and column, except for explorative improvisation and lean search. However, the discriminate validity of all blocks was also acceptable because the square root of the AVE values of explorative improvisation (0.754) and lean search (0.695) were only slightly smaller than the correlation coefficient of the row and column in which they were located (Bag et al., 2020).

**Table 3:** The results of discriminate validity (*N* = 203).

	1	2	3	4	5	6
1. EPI	**0.835**					
2. EPR	0.776^***^	**0.754**				
3. TH	0.389^***^	0.293^***^	**0.864**			
4. TU	0.531^***^	0.430^***^	0.546^***^	**0.740**		
5. MU	0.358^***^	0.271^***^	0.475^***^	0.607^***^	**0.765**	
6. LS	0.242^**^	0.484^***^	0.578^***^	0.621^***^	0.757^***^	**0.695**

Diagonal elements are the square roots of the AVE; The elements that appeared in lower left–left are the Pearson correlation coefficient between constructs; EPI, exploitative improvisation; EPR, explorative improvisation; TU, technology uncertainty; MU, market uncertainty; TH, entrepreneurial team heterogeneity; LS, lean search. Significance levels are given below.

** *p* < 0.01;

*** *p* < 0.001.

We also conducted CFA of the structural model to assess the discriminate validity, and the results (Table 4) showed that the baseline model of the six-factor model (*χ^2^* = 354.653, *df* = 194, *χ^2^/df* = 1.828, *CFI* = 0.941, *TLI* = 0.930, *RMSEA* = 0.064, and *SRMR* = 0.056) was superior to the five-factor model (EPI, EPR, LS, TU, and MU + TH), the four-factor model (EPI, EPR, LS, and TU + MU + TH), the three-factor model (EPI, EPR, and LS + TU + MU + TH), the two-factor model (EPI, and EPR + LS + TU + MU + TH), and the single-factor model (EPI + EPR + LS + TU + MU + TH). Finally, we used two indicators to evaluate the multicollinearity of this study. All correlation coefficients of each block in our study were below than 0.8, and all variance inflation factor (VIF) values were below than 10 (Table 5). Based on the above results, we considered that the discriminant validity of the constructs to be acceptable.

**Table 4:** The CFA results for the structural model.

Model	*χ^2^*	*df*	*χ^2^/df*	CFI	TLI	RMSEA	SRMR
Single-factor: (EPI + EPR + LS + TU + MU + TH)	1508.887	209	7.220	0.526	0.477	0.175	0.144
Two-factor: (EPI, EPR + LS + TU + MU + TH)	1151.995	208	5.538	0.656	0.618	0.150	0.139
Three-factor: (EPI, EPR, LS + TU + MU + TH)	802.630	206	3.900	0.783	0.756	0.119	0.103
Four-factor: (EPI, EPR, LS, TU + MU + TH)	708.868	203	3.491	0.816	0.790	0.111	0.100
Five-factor: (EPI, EPR, LS, TU, MU + TH)	449.478	199	2.261	0.909	0.894	0.079	0.064
Baseline model: (EPI, EPR, LS, TU, MU, TH)	354.653	194	1.828	0.941	0.930	0.056	0.064

EPI, exploitative improvisation; EPR, explorative improvisation; TU, technology uncertainty; MU, market uncertainty; TH, entrepreneurial team heterogeneity; LS, lean search.

**Table 5:** The results for regression analysis (*N* = 203).

Variables	Model 1	Model 2	Model 3	Model 4	Model 5	Model 6	Model 7	Model 8
Firm type	−0.050	−0.070	−0.055	−0.030	−0.012	−0.070	−0.017	−0.005
Industry	−0.077	−0.058	−0.022	−0.043	−0.024	−0.029	−0.030	−0.021
Firm size	−0.303^***^	−0.354^***^	−0.271^***^	−0.288^***^	−0.227^***^	−0.253^***^	−0.261^***^	−0.217^***^
Firm age	−0.059	−0.044	−0.030	−0.056	−0.053	−0.034	−0.059	−0.052
EPI (2.019)		0.024	−0.148^+^	−0.127	−0.162^*^	−0.178^+^	−0.101	−0.213^*^
EPR (1.935)		0.465^***^	0.463^***^	0.406^***^	0.446^***^	0.510^***^	0.374^***^	0.481^***^
AE(1.229)		0.161^*^	0.139^*^	0.143^*^	0.124^*^	−0.089	−0.046	0.077
TH (1.256)			0.386^***^			0.371^***^		
TU (1.324)				0.384^***^			0.397^***^	
MU (1.215)					0.483^***^			0.464^***^
EPI × TH						−0.009		
EPR × TH						0.111		
AE × TH						−0.211^*^		
EPI × TU							0.188^*^	
EPR × TU							0.093	
AE × TU							−0.003	
EPI × MU								−0.089
EPR × MU								0.192^*^
AE × MU								0.015
*R* ^2^	0.105	0.300	0.419	0.411	0.492	0.439	0.443	0.509
Adj-*R*^2^	0.087	0.275	0.395	0.387	0.472	0.409	0.411	0.481
∆*R*^2^		0.195	0.314	0.306	0.387	0.334	0.338	0.404
*F*-value	5.792^***^	11.952^***^	17.499^***^	16.943^***^	23.533^***^	13.610^***^	13.796^***^	18.017^***^

VIF values are in parentheses; EPI, exploitative improvisation; EPR, explorative improvisation; TU, technology uncertainty; MU, market uncertainty; TH, entrepreneurial team heterogeneity; LS, lean search. Significance levels are given below.

+ *p* < 0.1;

* *p* < 0.05;

*** *p* < 0.001.

### Hypothesis testing

We performed multiple regression analyses to test our hypotheses. The regression results are shown in Table 5: First, only the control variables were placed in model 1. H1, H2, and H3 proposed that exploitative, explorative, and ambidextrous improvisation were positively related to lean search. To test this, the estimated results of model 2 were used. We found that the regression equation was significant (*F* = 11.952, *p* = 0.000, *R*^2^ = 0.300), which suggested that exploitative, explorative, and ambidextrous improvisation explained 30.0% of the variation in lean search. Moreover, explorative improvisation (*β* = 0.465, *p* < 0.001) and ambidextrous improvisation (*β* = 0.161, *p* < 0.05) were both positively related to lean search. However, exploitative improvisation was not significantly related to lean search (*β* = 0.024, *p* > 0.1), suggesting that H1 was not supported, but H2 and H3 were supported.

In addition, to test H4a, H4b, and H4c, we synthesized the estimated results of model 2, model 3, and model 6 (Table 5). The results demonstrated that the regression equation (in model 6) was significant (*F* = 13.610, *p* = 0.000, *R*^2^ = 0.439), which indicated that improvisation (exploitative, explorative, and ambidextrous improvisation), team heterogeneity, and the interaction effect of improvisation and team heterogeneity could explain 43.9% of the variation in lean search. In the meantime, entrepreneurial team heterogeneity negatively moderated the relationship between ambidextrous improvisation and lean search (*β* = −0.211, *p* < 0.05). On the other hand, the moderating effects of entrepreneurial team heterogeneity were not significant in both aspects, i.e., the relationship of exploitative improvisation (*β* = −0.009, *p* > 0.1) and the explorative improvisation (*β* = 0.111, *p*>0.1) with lean search, which demonstrated that H4a, H4b, and H4c were not supported.

Similarly, to test H5a, H5b, and H5c which proposed that market uncertainty positively moderates the relationship between improvisation and lean search, we synthesized the estimated results of model 2, model 5, and model 8 (Table 5). The results showed that the regression equation (in model 8) was significant (*F* = 18.017, *p* = 0.000, *R*^2^ = 0.509), which suggested that improvisation (exploitative, explorative, and ambidextrous improvisation), market uncertainty, and the interaction effect of improvisation and market uncertainty explained 50.9% of the variation in lean search. Also, market uncertainty positively moderated the relationship between explorative improvisation and lean search (*β* = 0.192, *p* < 0.05). The moderating effects of market uncertainty on the relationship of exploitative improvisation (*β* = −0.089, *p* > 0.1) and ambidextrous improvisation (*β* = 0.015, *p* > 0.1) with lean search were not significant. Thus, H5b was supported, while H5a and H5c were not supported.

Finally, to test the positively moderating effects of technology uncertainty on the relationship between improvisation and lean search (H6a, H6b, and H6c), we synthesized the estimated results of model 2, model 4, and model 7 (Table 5). The results confirmed that the regression equation (in model 7) was significant (*F* = 13.796, *p* = 0.000, *R*^2^ = 0.443), which indicated that improvisation (exploitative, explorative, and ambidextrous improvisation), technology uncertainty, and the interaction effect of improvisation and technology uncertainty could explain 44.3% of the variation in lean search. Additionally, technology uncertainty positively moderated the relationship between exploitative improvisation and lean search (*β* = 0.188, *p* < 0.05). Nevertheless, the moderating effects of technology uncertainty on the relationship of explorative improvisation (*β* = 0.093, *p* > 0.1) and ambidextrous improvisation (*β* = −0.003, *p* > 0.1) with lean search were not significant. Thus, H6a was supported, while H6b and H6c were not supported.

**Figure 2:**
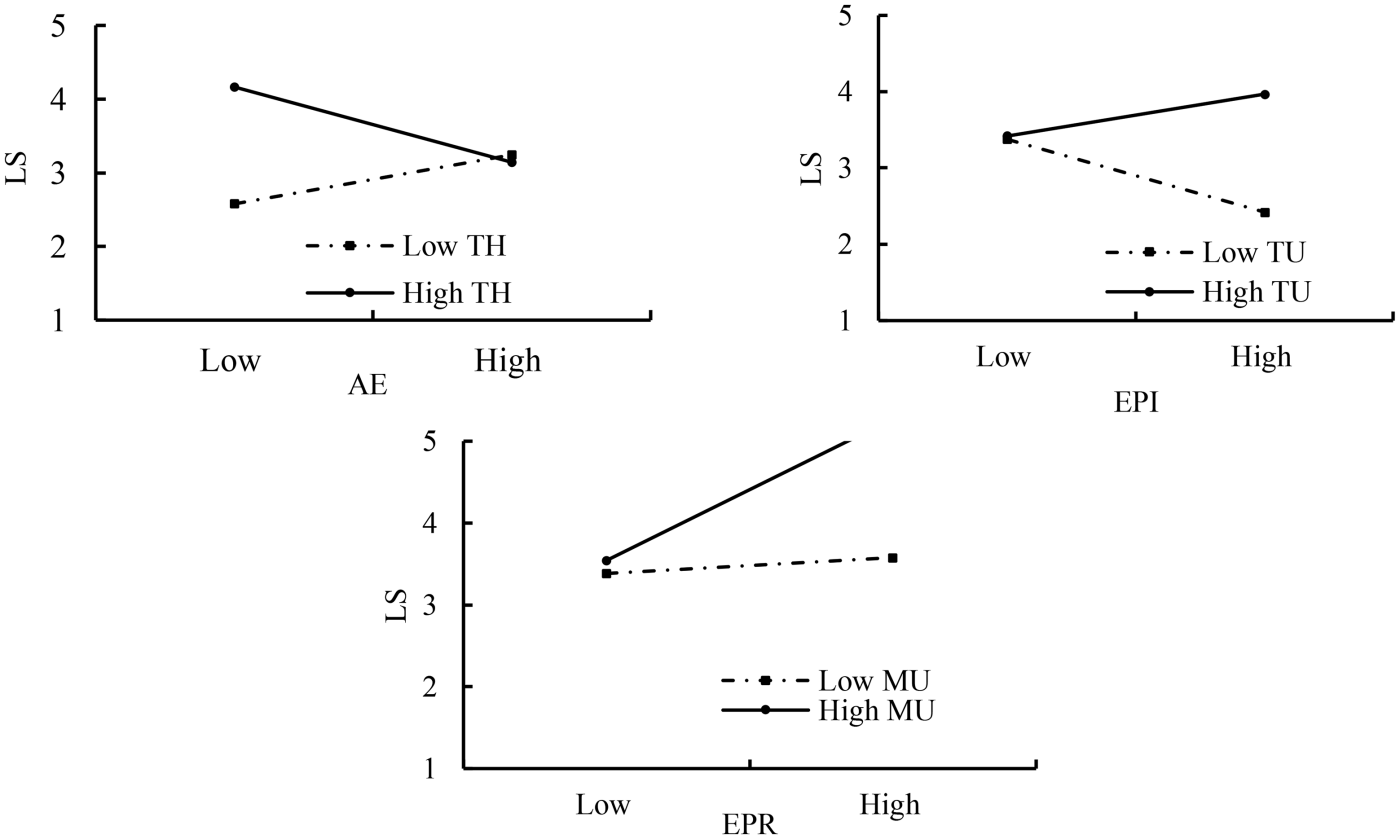
Moderating effects of entrepreneurial team heterogeneity and environmental uncertainty between improvisation and lean search. EPI, exploitative improvisation; EPR, explorative improvisation; TU, technology uncertainty; MU, market uncertainty; TH, entrepreneurial team heterogeneity; LS, lean search.

To clarify the direction of moderating, we plotted moderating effects of entrepreneurial team heterogeneity and environmental uncertainty (Figure 2).

## Discussion and conclusion

### Discussion of results

First, the study proves that explorative and ambidextrous improvisation are positively associated with lean search. However, the positive effect of exploitative improvisation on lean search is unsupported. These findings contribute to identifying the important antecedent factors of lean search in a rapidly changing context. Compared to the efficient utilization of existing resources, rapid acquisition and development of new resources are more conducive to helping startups in identifying and meeting customer demands in a short time. This is because explorative improvisation plays an important role in alleviating resource constraints and promoting organizational diversity (Ye and Mai, 2018). On the other hand, ambidextrous improvisation widens the scope and breadth of customer needs and product iterations by balancing the exploitive and explorative improvisation. This accelerates the flow, integration, and innovation of internal and external knowledge (Xiong et al., 2019). Nevertheless, the available resources might be used more for product optimization and innovation than for determining and satisfying customer demands. Therefore, the effect of exploitative improvisation on lean search is not obvious. Thus, this study provides empirical evidence to understand the relationship between improvisation and lean search from an ambidextrous perspective.

Second, the study confirms that the relationship between exploitative improvisation and lean search is positively moderated by technology uncertainty. However, the moderating effects of technology uncertainty between explorative, ambidextrous improvisation, and lean search are not significant. These findings indicate that although the rapid response to environmental changes is the primary reason for startups to conduct entrepreneurial activities, improving the efficient utilization of existing resources should be preferred to the innovation of new resources in the context of technological change. Thus, startups are likely to accelerate angel customer development based on existing resources (Ghezzi and Cavallo, 2020). However, the risks in the experimentation and innovation of new resources increase with the changes in technology. This might lead the startups to reduce the utilization of explorative and ambidextrous improvisation. Our findings support previously published results on the boundary conditions of lean search.

Thirdly, this study reveals that market uncertainty positively moderates the relationship between explorative improvisation and lean search. On the contrary, the moderating effects of market uncertainty are not significant between exploitative, ambidextrous improvisation, and lean search. These empirical results show that the instability of customer demands causes startups to pay greater attention to the innovative utilization of new resources. This is because innovation generally leads to the diversification of product development and business model design (Felin et al., 2020). It is useful to quickly identify and meet customer demands. However, exploitative improvisation is primarily a form of incremental innovation since it focuses on the efficient utilization of existing resources. Thus, the interaction between exploitative improvisation and market uncertainty is not obvious. These findings also contribute to extending the conditions under which lean startup occurs (Zhu and Dong, 2021).

Fourth, this study does not find the positive moderating effects of entrepreneurial team heterogeneity in the relationship between improvisation (exploitative, explorative, and ambidextrous improvisation) and lean search. Instead, entrepreneurial team heterogeneity negatively moderates the relationship between ambidextrous improvisation and lean search. These findings suggest that cognitive conflicts might be more obvious than cognitive diversities, which might reduce the efficiency of rapid decision-making in startups. The balance of resource management becomes more unfavorable with an increase in the degree of difference between the entrepreneurial teams. This can inhibit the activities of “customer positioning,” “customer development,” and “product iteration.”

### Theoretical contributions

This study has two theoretical contributions. First, this study conducts a pioneering work to link improvisation with lean search from an ambidextrous perspective. Our finding might narrow the gap that previous studies have ignored concerning the antecedent factors of lean search (Zhu and Dong, 2021). Previous studies primarily investigate the unique value of lean search. However, the ways to achieve it in a short time are not well-known. This study finds that both explorative and ambidextrous improvisation are positively related to lean search. However, there is no positive effect of exploitative improvisation on lean search. These findings contribute to enriching the managerial literature on improvisation and lean startup (Hmieleski and Corbett, 2006; Xiong et al., 2019; Shepherd and Gruber, 2021).

Second, this study also fills the gap that previous studies ignored concerning the boundary conditions of lean search. By considering entrepreneurial team heterogeneity and environmental uncertainty as core internal and external moderators, the study theoretically determines the conditions under which the lean search gets stronger or weaker. Specifically, technology uncertainty positively moderates the relationship between exploitative improvisation and lean search. On the other hand, market uncertainty positively moderates the relationship between explorative improvisation and lean search. However, entrepreneurial team heterogeneity has a negative moderating effect on the relationship between ambidextrous improvisation and lean search. Our findings enhance the understanding of the match between different types of improvisation and the different internal and external conditions to promote lean search.

### Managerial implications

This study has some interesting implications for practitioners. First, in order to realize lean search, startups should focus on improvisation to accelerate the “develop-test-learn” feedback loop in a rapidly changing environment. Compared to the utilization of existing resources, startups should focus on innovating the allocation of new resources at hand. Since the synergistic effect of exploitative and explorative improvisation contributes to achieving lean search, startups need to emphasize the interaction between existing and new resources.

Second, since technology uncertainty positively moderates the effect of exploitative improvisation on lean search, startups need to improve the utilization of existing resources to meet customer demands when they are in a highly changing technological environment. On the contrary, when industries are in high market uncertainty, startups should utilize new resources to facilitate lean search.

Third, startups need to establish a team with a consistent cognitive concept, educational background, and risk preference while conducting a lean search. They should rely on improvisation since entrepreneurial team heterogeneity might inhibit the process. Additionally, startups should also undertake regular communication projects in order to reduce the cognitive conflict between team members and improve the ability to quickly develop entrepreneurial activities.

### Limitations and future research

Like other empirical articles, this research has three limitations. First, although the study adopts several methods to ensure data quality, there is inevitably some subjective bias in the survey data (Ye and Mai, 2018; Yang et al., 2019). Therefore, future studies may perform situational experiments and collect longitudinal data to reduce data errors. Second, this study only discusses the antecedent factors of lean startup by considering improvisation. However, there might be other factors worth identifying, such as the types of entrepreneur cognition and business model design. Thus, future studies should explore these issues from a new perspective. Third, our study does not investigate each characteristic of entrepreneurial team heterogeneity, such as age heterogeneity and experience heterogeneity. Future studies might examine the moderating effects of different parameters.

## Data availability statement

The raw data supporting the conclusions of this article will be made available by the authors, without undue reservation.

## Author contributions

BH writing the manuscript and mentoring. JS planning the study and writing the manuscript. YJ reviewing the manuscript and providing language services. YX data analysis. YL providing the methodology. All authors contributed to the article and approved the submitted version.

## Funding

This research work was supported by the Fundamental Research Funds for the Central Universities (no. 2020CDJSK02PT12), the Natural Science Foundation of Chongqing (no. cstc2019jcyjmsxmX0616), the Graduate Research and Innovation Foundation of Chongqing, China (grant no. CYB20052), and the National Social Science Foundation of China (grant no.17XGL008).

## Conflict of interest

The authors declare that the research was conducted in the absence of any commercial or financial relationships that could be construed as a potential conflict of interest.

## Publisher’s note

All claims expressed in this article are solely those of the authors and do not necessarily represent those of their affiliated organizations, or those of the publisher, the editors and the reviewers. Any product that may be evaluated in this article, or claim that may be made by its manufacturer, is not guaranteed or endorsed by the publisher.

## Supplementary material

The Supplementary material for this article can be found online at: https://www.frontiersin.org/articles/10.3389/fpsyg.2022.940273/full#supplementary-material
