# Supplementary material for: How improvisation drives lean search: The moderating role of entrepreneurial team heterogeneity and environmental uncertainty
Source: Front Psychol. 2022 Sep 29;13:940273. doi: 10.3389/fpsyg.2022.940273 (PMC9557979; doi:10.3389/fpsyg.2022.940273)
Supplement: Supplementary file 1 [file Table_1.DOCX]

# APPENDIX

**Scale Items for Key Constructs**

To what extent do you agree with the following statements?

| **Constructs and Items** | **Resource** |
| --- | --- |
| *Exploitation Improvisation (1 = strongly disagree to 7 = strongly agree)* | Ye and Mai (2018) |
| 1. In product development, quickly develop relevant skills for immediate response. |  |
| 2. Fully exploit the neglected value of existing processes and production skills and deal with emergencies timely. |  |
| 3. Rapid integration of past experience for response action. |  |
| 4. Rapid improvement of existing products or processes to introduce new products and processes. |  |
| 5. Refine and extend existing product-related knowledge, skills and processes in real time. |  |
| *Exploration Improvisation (1 = strongly disagree to 7 = strongly agree)* | Ye and Mai (2018) |
| 1. Use the network at hand to quickly search for new production techniques and skills. |  |
| 2. Explore and acquire new technical information on the spot to construct coping strategies. |  |
| 3. Take a groundbreaking new approach that can be implemented with the resources at hand. |  |
| 4. Brainstorm new solutions or ways of working quickly and put them into action immediately. |  |
| 5. Develop new technologies or products immediately to achieve creative responses to new customer groups. |  |
| *Lean Search (1 = strongly disagree to 7 = strongly agree)* | Yang et al. (2019) |
| 1. Our marketing strategies are not immutable, on the contrary, they are very flexible. |  |
| 2. We do not know where their customers are at the beginning, but slowly explore to determine their target market. |  |
| 3. Our products are highly customized according to customer needs, but they are flexible in pricing. |  |
| 4. We attach great importance to customer feedback because it can help improve and optimize existing product processes. |  |
| 5. We pay great attention to the establishment of external relations, because most of the dealers are introduced by friends. |  |
| 6. When the product development is completed, we will set some key market indicators to check the product and customer demand matching degree, so as to formulate and optimize the new marketing strategy. |  |
| *Team heterogeneity (1 = strongly disagree to 7 = strongly agree)* | Narnjo-Gil (2009);  Talke et al. (2010) |
| 1. There is a wide age gap among members of corporate management teams. |  |
| 2. The tenure of management team members in the enterprise varies greatly. |  |
| 3. The educational background of management team members varies greatly. |  |
| 4. The professional backgrounds of management team members vary greatly. |  |
| 5. Management teams vary widely in their specialties. |  |
| *Environmental uncertainty (1 = strongly disagree to 7 = strongly agree)* | Jaworski and Kohli (1993);  Jiang and Ma (2018) |
| 1. Most new products on the market are made possible through technological breakthroughs. |  |
| 2. The core product technology of this industry changes rapidly |  |
| 3. The pace of technological change in the industry is very rapid |  |
| 4. Customer needs change rapidly |  |
| 5. Customer loyalty to products changes quickly |  |
| 6. Customers are always on the lookout for new products |  |
